# HLA-DR expression in melanoma: from misleading therapeutic target to potential immunotherapy biomarker

**DOI:** 10.3389/fimmu.2023.1285895

**Published:** 2024-01-17

**Authors:** Karim Amrane, Coline Le Meur, Benjamin Besse, Patrice Hemon, Pierre Le Noac’h, Olivier Pradier, Christian Berthou, Ronan Abgral, Arnaud Uguen

**Affiliations:** ^1^ Department of Oncology, Regional Hospital of Morlaix, Morlaix, France; ^2^ Inserm, Unité mixte de recherche (UMR1227), Lymphocytes B et Autoimmunité, Univ Brest, Inserm, LabEx Immunotherapy-Graft-Oncology (IGO), Brest, France; ^3^ Department of Radiotherapy, University Hospital of Brest, Brest, France; ^4^ Department of Cancer Medicine, Gustave Roussy Cancer Centre, Villejuif, France; ^5^ Faculty of Medicine, University Paris-Saclay, Le Kremlin Bicêtre, France; ^6^ Department of Pathology, University Hospital of Brest, Brest, France; ^7^ Department of Hematology, University Hospital of Brest, Brest, France; ^8^ Department of Nuclear Medicine, University Hospital of Brest, Brest, France; ^9^ UMR Inserm 1304 Groupe d'Étude de la Thrombose de Bretagne Occidentale (GETBO), IFR 148, University of Western Brittany, Brest, France

**Keywords:** melanoma, MHC-II, HLA-DR, immunotherapy, anti-PD1, BRAF, *NRAS*

## Abstract

Since the advent of anti-PD1 immune checkpoint inhibitor (ICI) immunotherapy, cutaneous melanoma has undergone a true revolution with prolonged survival, as available 5-year updates for progression-free survival and overall survival demonstrate a durable clinical benefit for melanoma patients receiving ICI. However, almost half of patients fail to respond to treatment, or relapse sooner or later after the initial response to therapy. Little is known about the reasons for these failures. The identification of biomarkers seems necessary to better understand this resistance. Among these biomarkers, HLA-DR, a component of MHC II and abnormally expressed in certain tumor types including melanoma for unknown reasons, seems to be an interesting marker. The aim of this review, prepared by an interdisciplinary group of experts, is to take stock of the current literature on the potential interest of HLA-DR expression in melanoma as a predictive biomarker of ICI outcome.

## 1 Introduction

Immunotherapy with immune checkpoint inhibitors (ICI) have revolutionized the treatment of patients with advanced solid cancers (1). Cutaneous melanoma (CM) is one of the most sensitive tumors to PD1 checkpoint inhibitors (nivolumab, pembrolizumab) (2).

Despite the paradigm shift brought about by ICI (prolonged survival and good tolerance (3–6)), 40 to 65% of metastatic melanomas do not respond to mono- or combo-ICIs and more than 43% of patients develop secondary resistance after a first response at 3 years of treatment (3).

The tumor microenvironment (TME) and the interactions between immune and non-immune tumor cells are of crucial importance in cancer initiation and progression, for example by delivering extracellular signals that support and promote peripheral immune tolerance (7).

Among the components of this TME is Human Leukocyte Antigen – DR isotype (HLA-DR), which is expressed on professional antigen-presenting cells (pAPCs) and unexplainedly on non-pAPC cells such as certain tumors, and in greater proportion in melanoma (8, 9).

In this article, we first present an overview of HLA-DR with its role in the tumor cell as well as its interaction with TME before reviewing studies evaluating the response to ICI in melanoma based on HLA-DR expression and, finally, we discuss how HLA-DR could fit into therapeutic application as a biomarker.

## 2 HLA-DR: role and interaction with tumor microenvironment

The efficacy of ICI immunotherapy depends on the recognition of the antigens by T cells. This recognition is mediated by the major histocompatibility complex (MHC) molecules that present the antigens to the T cell receptor (TCR), with these interactions being increased by co-receptors such as CD4 on helper T cells and CD8 on cytotoxic T cells. MHC class I molecules (MHC-I) are expressed by most nucleated cells and mainly present peptide antigens of endogenous origin to CD8+ T cells. MHC class II (MHC-II) molecules are mostly expressed by professional antigen-presenting cells (PAPCs) such as dendritic cells (DCs), B cells and macrophages, and mainly present peptide antigens of exogenous origin to CD4+ T cells. Among the MHC-II components, HLA-DR is the most frequently expressed and the most studied (10, 11). HLA-DR is encoded by the human leukocyte antigen complex on the region 6p21.31 of chromosome 6 (12). HLA-DR is composed of two non-covalently associated transmembrane glycoproteins (the α and β chains) (13, 14), and is primarily expressed on B lymphocytes, monocytes, dendritic cells and thymic epithelial cells. In addition to hematopoietic-lineage neoplasia, HLA-DR is likewise expressed by certain solid tumors, including malignant CM, lung cancer, liver, cancer, glioblastoma, renal cancer (8).

To date, no relationship between HLA-DR expression and the aggressiveness of most tumors or their prognostic factors has been noted in most of the different tumor types although in CM, an association between HLA-DR expression and the metastatic and aggressive potential of the disease was initially suggested (15–17).This assertion was later challenged by finding no particular impact on the aggressive character of CM (9, 18).

The function of MHC-II expression in tumor cells has long been unknown; recently, several studies have demonstrated that CD4 T cells can recognize melanoma cells in an antigen-specific, MHC class II-dependent manner (19–21).

**Figure 1:**
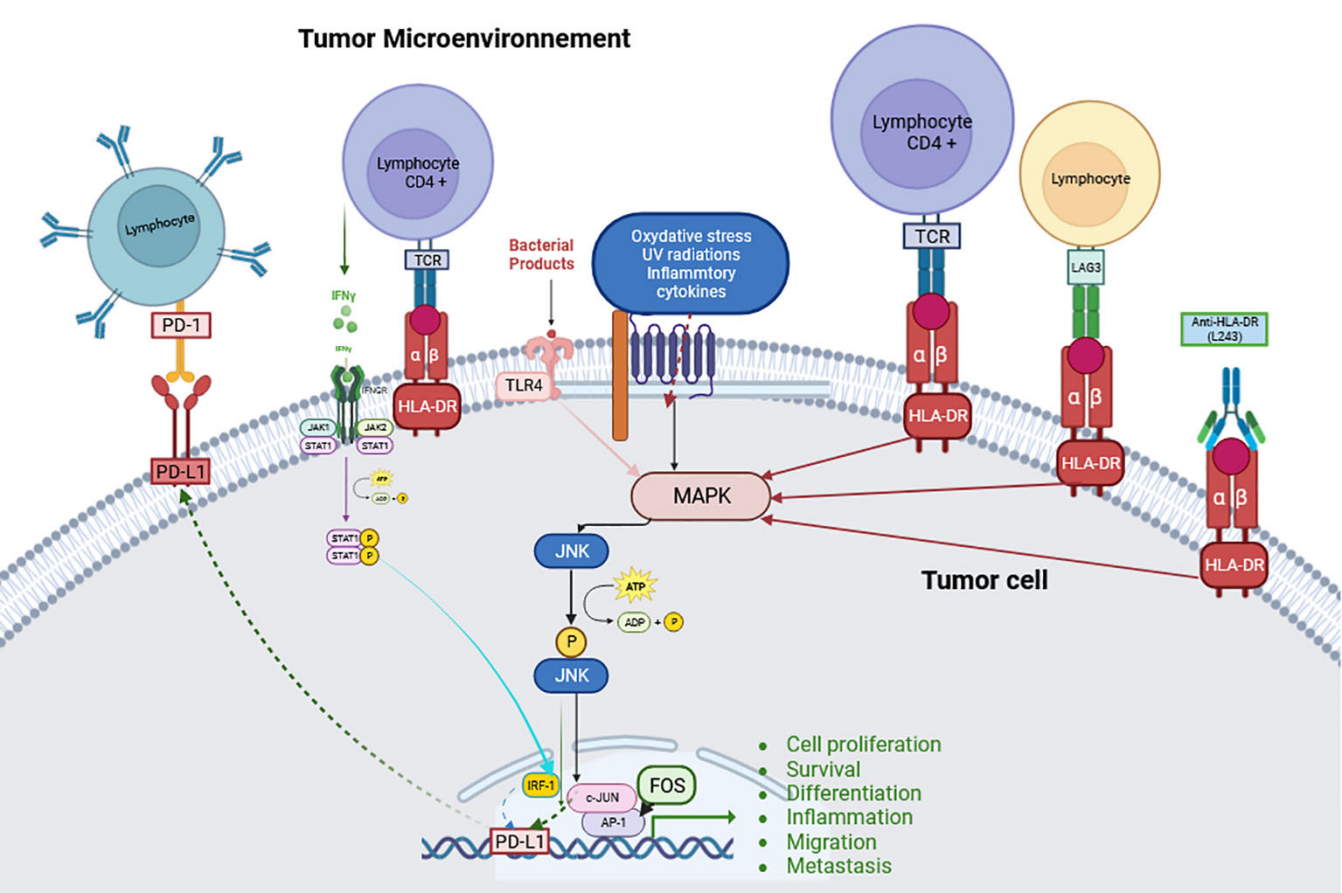
Schematic explanation of the JNK pathway and its signaling with HLA-DR. (Created with BioRender.com) JNK can be activated by a series of stimuli via specific MAP3Ks. This activation allows transcription of various downstream targets for tumorigenesis events such as cell proliferation, survival, differentiation, inflammation, migration, metastasis and immune evasion such as PD-L1 transcription (Abbreviations: defined in the main text).

In solid tumors, HLA-DR has been predominantly studied in CM. Based on the results obtained - after induction using high concentrations of the specific anti-HLA-DR monoclonal antibody L243- *in vitro* in cell lines without *in vivo* confirmation, it appears that tumor cells growth and aggressiveness may be due to HLA-DR-mediated signaling that induces ILK/AKT (integrin-linked kinase/protein kinase B), FAK/PAX/AKT (focal adhesion kinase (FAK)/paxillin/Protein kinase B) and BRAF/ERK (extracellular signal-related kinases) signaling pathways activation as well as the lipid rafts recruitment of FAK and AKT proteins (22–25). Constantini et al. have demonstrated *in vitro* in cell lines that HLA-DR expression, through these signaling platforms, modulates the interaction of melanoma cells with the microenvironment that is considered crucial for their metastatic dissemination. MHC-II mediated signaling, including HLA-DR, increases the expression of integrins and cell adhesion molecule (CAM) receptors, activating associated signaling and enhancing melanoma cell motility and invasiveness. This signaling also modulates multiple intracellular processes associated with cell invasion based on increased integrins function. In addition, signal transducer and activator of transcription 3 (STAT3), mitogen-activated protein kinase (MAPK) and phosphatidylinositol 3-kinase (PI3K)/AKT signaling pathways activate the expression of PD-L1 receptor, which contributes to melanoma immune escape (25) (**Figure 1**).

Hemon et al. have shown *in vitro* in cell lines that LAG-3 can, in addition to activating the PI3K/Akt pathway, also activate the MAPK/Erk pathway (26) like the anti-HLA-DR antibody L243 (which only activates the MAPK/Erk pathway), but with different kinetics (24) (**Figure 1**). Also based on the *in vitro* study of the A375 line, expressing HLA-DR, Barbieri et al. demonstrated via stimulation with the anti-HLA-DR antibody (L243), that the interaction between HLA-DR and the TCR leads to the activation of c-Jun N-terminal kinase (JNK), a member of the MAPK family which plays an essential role in regulation of cell proliferation, metabolism, survival and death, and of DNA repair, but with no evidence that induction of this TCR CD4+ signaling would lead to an effect similar to that previously reported on activation of the MAPK/Erk pathway (27). Thus, JNK activation had been shown to promote tumor proliferation, as demonstrated in glioblastoma (28), non-small cell lung cancer (NSCLC) (29) and pancreatic cancer (30, 31), or to immune evasion as in breast (32) and oropharynx cancers (33). The role of this kinase is critical in tumor growth and progression, as phosphorylated JNK dimerizes Jun proteins, particularly c-Jun with Fos proteins (c-Fos, FosB, Fra-1, and Fra-2) to form AP-1 (33). AP-1 is then involved in cell proliferation, survival, differentiation, inflammation, migration, and metastasis (34) (**Figure 1**). JNK contributes to immune evasion via PD-L1 expression by modulating the activity of c-Jun, an inducible transcription factor that directs gene expression changes such as PD-L1, a mechanism observed in melanoma (35); or via TLR4 (toll-like receptor 4) signaling as in bladder cancer (36) (**Figure 1**).

In addition, HLA-DR may be involved in immune evasion, as Oliviera et al. have identified three general types of potential interactions between tumor-specific CD4+ tumor-infiltrating lymphocytes (TILs) cells and melanoma in a cohort of CM. One of these mechanisms strongly implicates MHC-II/HLA-DR. The authors demonstrated the direct tumor specificity of over 70% of the TCRs generated in the TME of MHC-II/HLA-DR melanomas (2/4 patients). The majority of these TCRs showed specificity for neoantigens with avidities similar to those of exhausted lymphocyte TCRs, suggesting that their stimulation could lead to the activation of immunosuppressive regulatory lymphocytes CD4+ (Treg). The authors also found that MHC-II/HLA-DR melanomas were characterized by high numbers of CD8+ TILs, due to their association with extreme tumor mutational burden (TMB). In these conditions, the reactivation of CD8+ responses can disrupt the balance between effector and Treg cells, thus favoring the high immunogenicity expected of MHC-II/HLA-DR melanomas (37).

Furthermore, Donia et al. highlighted a new mechanism of immune escape, in an analysis of a cohort of 38 patients, 50% of whom had native MHC-II expression. Tumor-specific CD4+ T cell responses were dominated by tumor necrosis factor (TNF) production. Chronic exposure to local TNF reduced CD8+ T cell activation in Interferon-γ (IFN-γ)-rich TME. Conversely, direct CD4+ T cell responses had no effect on melanoma cell proliferation or viability (38).

MHC-II shares several characteristics with other tumor-associated immunosuppressive molecules, such as Indoleamine 2,3-dioxygenase (IDO) and PD-L1. Indeed, MHC-II is aberrantly activated in some melanomas and, exactly like IDO and PD-L1 (38, 39), is upregulated by IFN-γ-mediated immune responses. Thus, *in situ* detection of MHC class II in melanoma may represent constitutive expression in CM cells or be induced by the presence of IFN-γ-secreting cells (e.g. tumor antigen-specific CD8+ T cells), or both. Interestingly, CD4+ T binding to MHC-II-positive tumor cells induces IFN-γ secretion (40), which is a potent inducer of PD-L1 (41–43) (**Figure 1**).

Finally, HLA-DR is also an immune control point, as it is the ligand for lymphocyte-activation gene 3 (LAG3) (44), which is present on the surface of T cells, NK cells and plasmacytoid dendritic cells (45). The LAG3 protein forms a stable link to HLA class II through its 30-amino acid loop structure, and selectively binds to peptide-containing MHC-II (44, 46). Under normal circumstances, LAG3 can help prevent autoimmune responses or excessive responses against viral infections (44). However, tumor cells can use immune checkpoints to avoid immune recognition and deplete cytotoxic T cells. LAG3 is strongly associated and synergistic with PD-1 as it is co-expressed with this immune checkpoint on CD4 and CD8 T cells which blocks the anti-tumor immune response (47). LAG3 may also be a marker of immune exhaustion, which could be a factor in resistance to anti-PD-1 and anti-CTLA-4 (48).

The aim of the present paper is to review the current literature on the potential interest of HLA-DR expression in melanoma as a predictive biomarker of response to ICI.

## 3 Melanoma HLA-DR expression and response to ICI: literature analysis

HLA-DR is normally expressed in professional antigen-presenting cells but also on some tumor cells of certain tumors without the explanation of this expression being elucidated at the moment.

Well before the advent of ICI, the concept of immunotherapy was first introduced with the use of BCG in urothelial bladder cancer showing proven efficacy. Based on this finding, Brocker et al. (49) evaluated BCG injections as adjuvant therapy in a population of 107 patients with high-risk stage I melanoma. In this study, 44/107 (41%) of them had been treated with BCG. HLA-DR expression was assessed by immunohistochemistry with 910 D7, OKIal (Ortho Diagnostics), 12 (Coulter Electronics), anti HLA-DR (Becton Dickinson) and D 1 - 12 (Dr. S. Carrel, Lausanne) clones. Authors calculated the percentage of stained tumor cells and then grouped tumors according to their “low” (0-19% tumor cells positive/section) or “high” (20%-100% cells positive/section) HLA-DR expression. They found that HLA-DR expression was associated with a poor prognosis (p < 0.01) and no statistically significant benefit from BCG treatment, although there was a trend toward better progression-free survival (PFS) in BCG-treated patients not expressing HLA-DR.

At the current era of ICI therapies, different studies have evaluated the response to treatment according to HLA-DR status in solid tumor. It concerns mainly CM but also in only two other series non-small cell lung cancers (NSCLC) and urothelial carcinomas.

Regarding CM, first review of literature reported that HLA-DR expression was predictive of better survival and response to ICI, and was also associated with PD-L1 expression (25). As an illustration, Johnson et al. showed in their study that HLA-DR expression was required for anti-PD-1/PD-L1 activity. Indeed, they demonstrated in the first step of their study on 60 cell lines, that HLA-DR expression by melanoma-cells was associated with unique inflammatory signals that are more responsive to PD-1-targeted therapy (9). Afterwards, they studied HLA-DR expression on melanoma tumor tissue from 67 patients, including 53 metastatic disease (83%). HLA-DR expression by melanoma-cells was observed in 30.3% (20/67) of patients and tended to be more frequent (p = 0.47) in the *NRAS* mutated group (43%, 6/14) than in respectively the *BRAF* mutated group (23%, 3/13) and the *BRAF/NRAS* wild-type group (28%, 11/39). Among 30 patients with metastatic disease treated by ICI (anti-PD1/anti-PDL1 and anti-CTLA4) HLA-DR expression in pre-ICI melanoma samples was quantified by 2 independent pathologists as tumors < 5% of HLA-DR positive melanoma cells (termed HLA-DR- with no significant expression, 16/30; 53.3% of patients) and tumors with > 5% of HLA-DR positive melanoma cells (termed HLA-DR+ with significant HLA-DR expression, 14/30; 46.7% of patients). The objective response rate (ORR) was significantly higher in the HLA-DR+ group than in the HLA-DR- group (79% versus 38% respectively, p = 0.033). These results were confirmed in a second independent external cohort of 23 melanoma treated with ICI (anti-PD-1). Indeed, the reported ORR in the HLA-DR+ group was 75% (6/8) versus only 27% (4/15) in the HLA-DR- group (p = 0.025). Interestingly, responders had clinico-biological factors of poor prognosis in this series such as bulky diseases, liver metastases and elevated Lactate Dehydrogenase (LDH) seric levels. Based on a 5% HLA-DR positive melanoma cells threshold, PFS was superior in the HLA-DR+ group (median not reached versus 3.2 months, p = 0.02) as well as OS (median not reached versus 27.5 months, p = 0.003). Similar results were found using HLA-DR positive melanoma cells thresholds of 1%, 10% and 20%. Of note, in a small group of 13 patients treated exclusively with anti-CTLA4 (ipilimumab), there was no significant association between therapeutic response and HLA-DR expression (9).

In another retrospective study, starting from the hypothesis that PD-1 and PD-L1 receptors must be expressed sufficiently for patients to respond to anti-PD-1 ICI, Johnson et al. searched correlation between expressions of PD-1, PD-L1, HLA-DR (anti-HLA-DR clone TAL.1B5, DAKO) and IDO and treatment responses of metastatic CM to pembrolizumab or nivolumab (50). In a first exploratory cohort of 24 patients from their medical center, authors showed that response to anti-PD1 was correlated with a high expression of IDO-1+/HLA-DR+ cells (5% threshold of positivity) with a sensitivity of 85%, a specificity of 91% and an area under the curve (AUC) of 0.88; whereas the biomarkers taken separately or with another combination did not allow to differentiate responders from non-responders. In a subsequent validation cohort of 142 patients from 10 medical centers, the authors observed higher response rates in patients with high PD1/PDL1 (p = 0.06) or IDO-1/HLA-DR expression scores (p = 0.0002). They also showed significantly improved PFS (HR = 0.36; p = 0.0004) and OS (HR = 0.39; p = 0.0011) in patients with high PD1/PDL1 and/or IDO-1/HLA-DR scores. Furthermore, multivariate analysis revealed that survival predictions were not influenced by commonly used clinico-biological factors, such as metastatic stage or LDH levels (p = NS), in contrast to biomarker signature (PD1/PDL1 or IDO-1/HLA-DR) (PFS with biomarker signature alone HR = 0.36 [0.20-0.65] (p = 0.00065); OS with biomarker signature alone: HR = 0.39 [0.21-0.70] (p = 0.0016). In addition, PD-L1 expression alone at any threshold (1%, 5%, or 25%) did not significantly (p > 0.1) identify patients with better PFS or OS, reinforcing the imperfection of this widely used biomarker prior to anti-PD-1/PD-L1 therapeutic decision outside the field of metastatic CM (51–54). The same authors later suggested that the immune resistance continuum of IFN-γ-mediated expression of PDL1, HLA-DR and IDO-1 results from PDL1/PD1 and HLA-DR/LAG3 interaction (55, 56).

Furthermore, an ancillary study to CheckMate 064 (sequential administration of nivolumab followed by ipilimumab, or the reverse sequence) and CheckMate 069 (nivolumab plus ipilimumab versus ipilimumab alone), has evaluated MHC-I and MHC-II protein expression in pre-treatment biopsy samples of untreated advanced melanoma. Analysis was performed in subgroups categorized as treated with ipilimumab followed by nivolumab (IPILIMUMAB→NIVOLUMAB), nivolumab followed by ipilimumamb (NIVOLUMAB →IPILIMUMAB), ipilimumab alone (IPILIMUMAB), or combination of both nivolumab and ipilimumab simultaneously (NIVOLUMAB+IPILIMUMAB) in the 2 clinical trials mentioned above (57). In CheckMate 064, IHC revealed that more than 1% of melanoma cells expressed MHC-II in 26/92 cases (28%). Otherwise, MHC-II positive melanoma cells were concentrated at the inflammatory and invasive margin of the tumor, that was consistent with induced local expression of MHC-II. The proportion of cases with >1% of MHC-II positive melanoma cells was quite similar in CheckMate 069 (29/89 cases, 33%). The authors found that MHC-II positivity (>1%) in CheckMate 064 was associated with a significant better outcome in subgroup NIVOLUMAB→IPILIMUMAB (p = 0.005) compared with the IPILIMUMAB→NIVOLUMAB subgroup (p = 0.31). This finding was consistent with the Johnson et al. study above-mentioned (9).

Then, in another study on 60 samples of CM before ICI initiation, in increasing the multiplexing of their immunotyping analyses until 44 markers, the authors further reported that HLA-DR expression in melanoma cells was both correlated with PFS (HR = 0.49; p = 0.0281) and OS (HR = 0.27; p = 0.0035) (58).

From the results of these studies, HLA-DR expression on melanoma cells could be an indicator of IFN-gamma release due to an ongoing anti-tumor immune response.

Finally, few studies have evaluated HLA-DR expression on tumoral microenvironment (TME) cells of melanoma and ICI therapy. In addition, no other type of solid tumor has been published on the subject.

A prospective phase Ib/II study had evaluated efficacy of the combination of pembrolizumab and high-dose interferon alfa-2b in 30 patients with resectable locally advanced melanoma in neoadjuvant strategy (59). The authors analyzed the composition of the TME before and after surgery with IHC on the pre- and post-surgery samples of 13 patients with residual pathological disease. Treatment response was associated with a significant increase in the percentage of CD8 T cells (p = 0.04) in the TME. It was also associated with a significant increase in both PD-1 (p = 0.04) and PD-L1 expression (p = 0.02) in non-tumor cells, and in PD-1/PD-L1 interaction (p = 0.008). But tumor cells expressing IDO1 and HLA-DR+ did not change significantly after treatment (p = 0.2). In another sub-analysis of 14 samples (5 with pathological complete response (pCR) and 9 without), high baseline HLA-DR values on non-tumor cells were associated with pCR (p = 0.008) in this cohort.

## 4 HLA-DR as a potential therapeutic target?

Although HLA-DR has shown interesting potential for predicting response to ICI and participating in the definition of hot-immune group, several limitations must nevertheless be noted.

There is no clear consensus on the antibody used for IHC, although LN3 appears to be the most sensitive and specific (60). The threshold for significant positivity is unclear: in the series studied, the 5% threshold had been used to establish the significance of HLA-DR to the therapeutic response to ICI. Although similar results were obtained using HLA-DR-positive melanoma cell thresholds of 1%, 10% and 20%, further studies with larger numbers could nevertheless clarify this point (9).

The HLA-DR antigen triggers signal transduction via the ILK/AKT, FAK/PAX/AKT and BRAF/ERK signaling pathways (61) with an action on the nuclear transcription factor AP-1 involved in cell proliferation and invasiveness (62). With such a background, HLA-DR blockade may be an interesting therapeutic target.

First, Altomonte et al. showed *in vitro* that HLA-DR blockade by L243 antibody induced a significant (p < 0.05) and dose-dependent growth inhibition of Mel120 metastatic melanoma cell line as well as their homotypic aggregation (63). To date, no anti-HLA-DR therapy has been tested in melanoma or other solid tumors.

However, some HLA-DR therapies have been developed in hematological malignancies, mainly in chronic lymphocytic leukemia (CLL) and lymphoma. The most promising molecule is apolizumab which is an IgG1 anti-1D10. The 1D10 antigen is a polymorphic determinant of the β chain of HLA-DR and its expression appears to be variable in humans as approximately 80% of healthy subjects express it. This antigen is expressed primarily on antigen-presenting cells, including B cells, monocytes and dendritic cells, and to a much lesser extent on some activated T cells and mesenchymal cells. It has been reported that B cells express it at the highest levels (64). The IgG1 1D10 is capable of inducing antibody-dependent cell-mediated cytotoxicity (ADCC), complement-dependent cytotoxicity (CDC) and direct apoptosis of 1D10 antigen-positive malignant B cells (64).

Three clinical trials have been initiated in humans, following interesting and promising results in rhesus monkeys with an acceptable safety profile, except for a type I hypersensitivity reaction that was adequately controlled by slow injection and anti-histamine premedication (65). First, a phase I trial in non-Hodgkin’s lymphoma (66) was conducted with apolizumab in combination with filgrastim to increase neutrophil counts and stimulate IgG-mediated ADCC activity. Results were disappointing as a PFS of 5.0 months was found after the first injection and a significant hematoxicity was seen in almost all patients (e.g. grade IV thrombocytopenia). However, due to the small cohort size (n=6), it was not possible to correlate 1D10 expression levels with clinical toxicity. The second was a phase I/II trial evaluating apolizumab in refractory CLL in 23 patients with apolizumab dose escalation 3 times per week (1.5, 3.0, 5.0 mg/kg/dose) for 4 weeks. The limiting toxic dose (DLT) was manifested by aseptic meningitis and hemolytic uremia syndrome (HUS) (67).

However, the combination of apolizumab and rituximab appears to be more effective than apolizumab alone. The reported AEs were similar to previous published series, with a mild yet manageable infusion reaction observed in the early cycles of treatment; and HUS was thus reported as a DLT (68).

Although targeting HLA-DR seems attractive due to its major signaling and activity profile observed with apolizumab, it appears difficult to envisage a clinical development at this time given the limiting toxicity due in part to the expression of HLA-DR in normal tissue.

Although targeting HLA-DR seems attractive because of the important signaling and activity profile observed with apolizumab, it seems difficult to consider for clinical development at this time. This is due in part to HLA-DR expression in normal tissues, but to its deleterious effect on the anti-tumor response. Oh et al. investigated the role of TCD4+ in bladder cancer and identified a perforin and granzyme mediated cytotoxic TCD4+ population. This TCD4+ population specifically targets MHC II-expressing tumor cells (69). This result has also been observed in melanoma (19). This mechanism could make HLA-DR inhibition potentially counterproductive.

Even if there are no therapies directly targeting HLA-DR in solid oncology, checkpoint inhibitors targeting its ligand, LAG3, have been developed in recent years. Among LAG3 inhibitors, 3 are in advanced development: ieramilimab from Novartis Lab (70), favezelimab from MSD Lab (71), and relatlimab from Bristol Myers Squibb Lab (72). These 3 Ac are IgG4.

In monotherapy, their activity is very modest: in a phase I trial evaluating ieramilimab in a population of 134 pre-treated patients, the objective response rate was 0%, with a SD assessed at 23.9%; the same applies to favezelimab, evaluated in a population of 20 pre-treated patients. However, relatlimab showed a response rate of 11.4% in a population of 68 pretreated patients (48). Nevertheless, the combination with an anti-PD1 appears more promising and may confirm the hypothesis of LAG3 is a marker of immune exhaustion, which could be a factor in resistance to anti-PD-1 and anti-CTLA-4 (48). In fact, in pre-treated patient populations, the combination with an anti-PD1 resulted in ORRs of 6.3% for favezelimab-pembrolizumab (71), 10.8% for ieramilimab-spartalizumab (70) and 44% for relatlimab-nivolumab (72). It should be noted that none of these studies assessed the status and quantification of LAG3, but not HLA-DR. Of the 3 molecules developed, only relatlimab combined with nivolumab has been approved as a first-line treatment by both the U.S. Food and Drug Administration (73) and the European Medicine Agency, which has restricted the indication to patients with tumor cell PD-L1 expression < 1% (74).

Finally, new antibodies combining anti-PD1 and anti-LAG3 on the same IgG are under development, with results that seem more promising than with separate antibodies (75).

## 5 Conclusion

Although HLA-DR can induce a signaling cascade leading to cell proliferation, its direct therapeutic targeting seems irrelevant due to its ubiquitous expression and the toxicity it may generate.

HLA-DR is a biomarker that was studied extensively in oncology during the 1980s before being neglected. Since the advent of immunotherapy, its interest has become essential to predict response. In addition to its involvement in the definition of the hot-immune group, the expression of HLA-DR in the tumor microenvironment, both on tumor and non-tumor cells, conditions the action of anti-PD1 checkpoint inhibitors and probably of the new checkpoint inhibitors under development.

## Author contributions

KA: Conceptualization, Data curation, Methodology, Resources, Software, Writing – original draft, Writing – review & editing. CL: Investigation, Methodology, Resources, Supervision, Writing – original draft. BB: Formal analysis, Project administration, Writing – review & editing. PH: Writing – review & editing. PL: Investigation, Writing – review & editing. OP: Investigation, Writing – review & editing. CB: Conceptualization, Project administration, Visualization, Writing – review & editing. RA: Conceptualization, Methodology, Project administration, Resources, Validation, Writing – review & editing. AU: Conceptualization, Project administration, Writing – review & editing.
